# Supplementary figures and images for: Interplay between UNG and AID governs intratumoral heterogeneity in mature B cell lymphoma
Source: PLoS Genet. 2020 Dec 23;16(12):e1008960. doi: 10.1371/journal.pgen.1008960 (PMC7790409; doi:10.1371/journal.pgen.1008960)

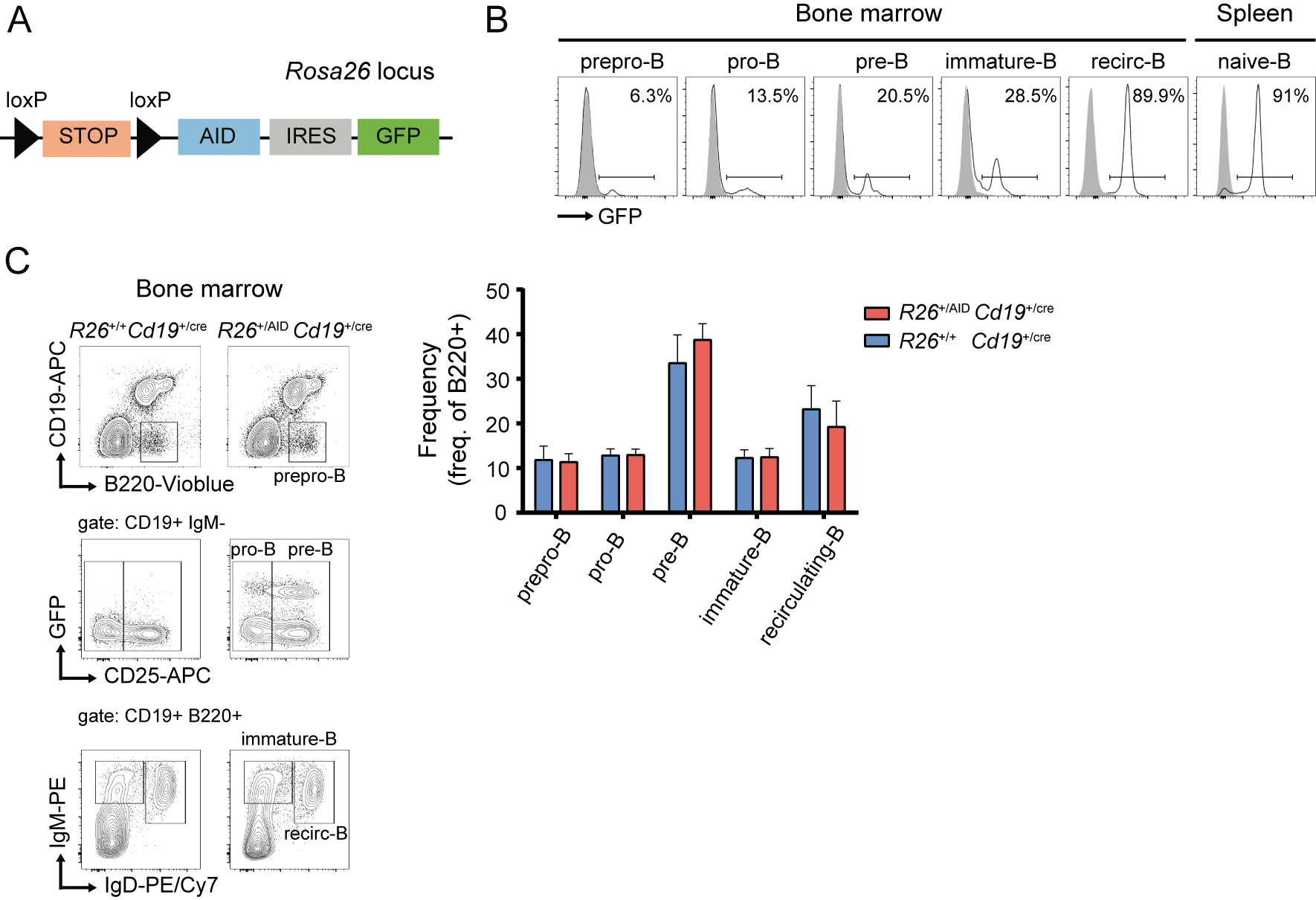

Supplement: S1 Fig — (A) Representation of the cassette used to generate the R26+/AIDCd19+/Cre mouse model. An AID-IRES-GFP construct preceded by a transcriptional STOP flanked by loxP sites was inserted within the Rosa26 locus. R26+/AID mice were then crossed with Cd19+/Cre mice to generate R26+/AIDCd19+/Cre mice. (B) Progressive R26-AID allele expression during B cell differentiation. Representative FACS analysis of GFP reporter expression in bone marrow and splenic B cells from R26+/AIDCd19+/Cre (black empty line) and R26+/+Cd19+/Cre (grey shade) mice. Bone marrow B cell subsets were gated as prepro (B220+CD19-), pro (B220+CD19+IgM-CD25-), pre (B220+CD19+IgM-CD25+), immature (B220+CD19+IgM+IgD-) and recirculating (B220+CD19+IgM+IgD+). Spleen B cells were gated as B220+. (C) B cells from R26+/AIDCd19+/Cre mice develop normally. Left, representative FACS plots of prepro, pro, pre immature and recirculating B cells from R26+/AIDCd19+/Cre and control R26+/+Cd19+/Cre mice. Right, frequency quantification of each B cell subpopulation in R26+/AIDCd19+/Cre (n = 9) and R26+/+Cd19+/Cre mice (n = 8). Two-tailed t-test, error bars represent SD. (TIF) [file pgen.1008960.s001.tif]

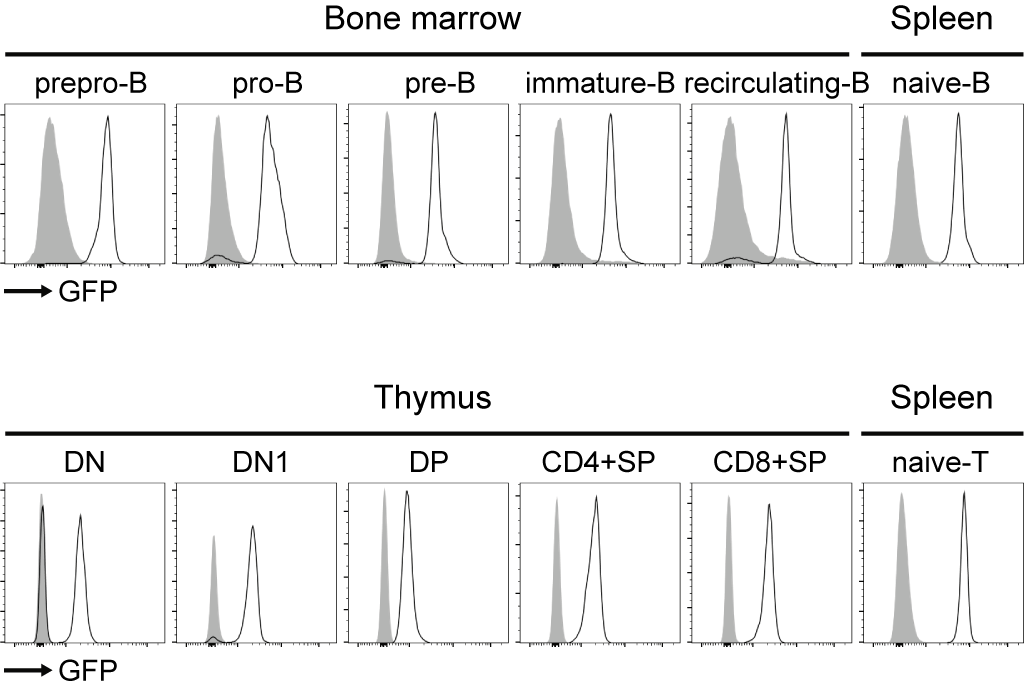

Supplement: S2 Fig — FACS analysis of GFP reporter expression in bone marrow, spleen and thymus cells from R26+/AIDVav+/cre (black empty line) and R26+/+Vav+/cre (grey shade). Bone marrow and spleen B cell subsets were gated as in S1B–S1C Fig. T cell subsets were gated as DN (CD4-CD8-), DN1 (CD4-CD8-CD44+CD25-), DP (CD4+CD8+), CD4+SP (CD4+CD8-), CD8+SP (CD4-CD8+) from thymus and naive-T (B220-CD3+) from spleen. (TIF) [file pgen.1008960.s002.tif]

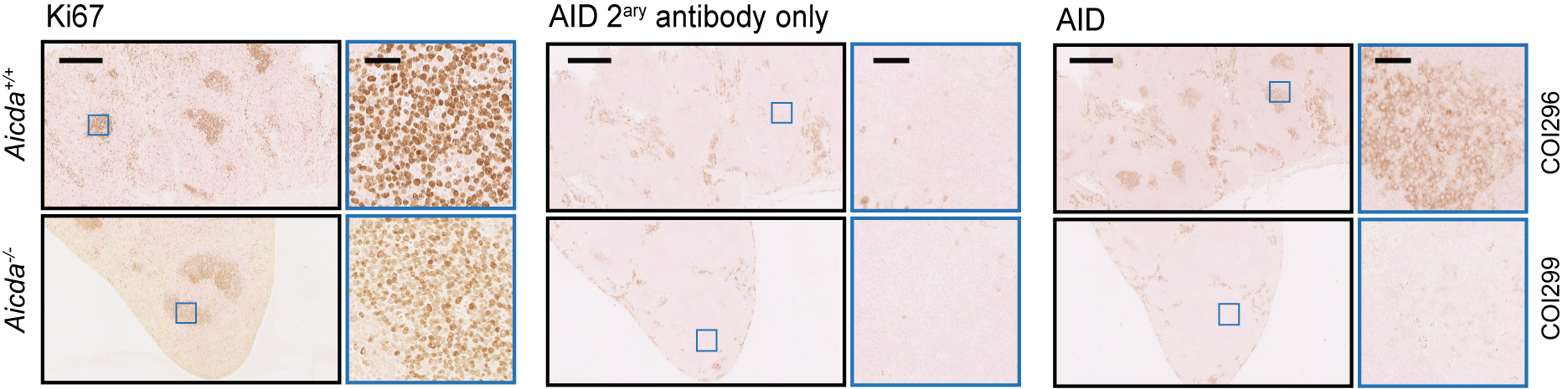

Supplement: S3 Fig — Ki67 and AID immunohistochemistry of spleen sections from SRBC immunized Aicda+/+ and Aicda-/- mice. Magnification is 5x inset is 40x. Scale bars are 500μm and 50μm for 5x and 40x images respectively. (TIF) [file pgen.1008960.s003.tif]

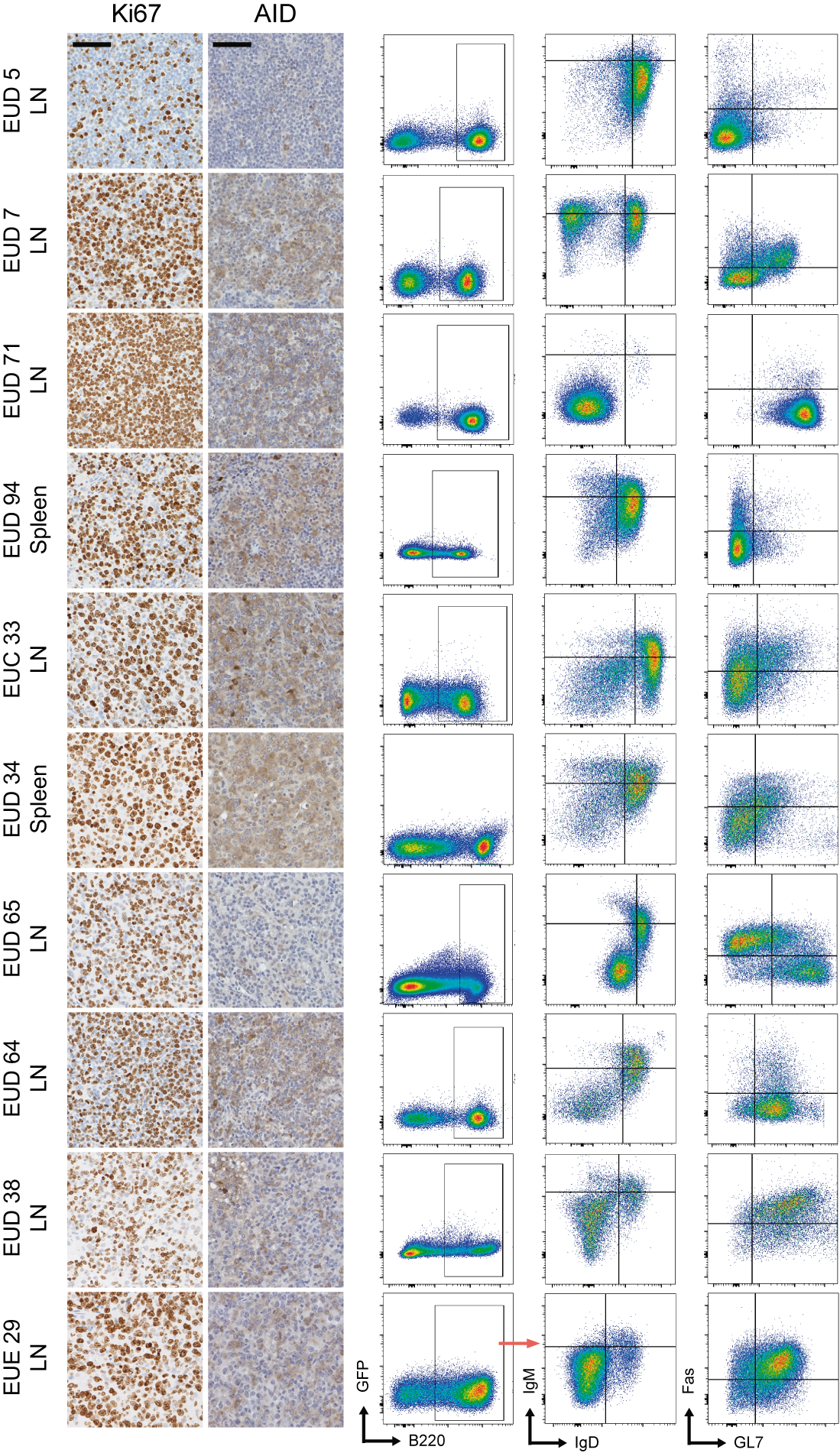

Supplement: S4 Fig — Magnification for Ki67 and AID IHQ images is 40x. Scale bar is 50μm. Total live cells (left FACs panel) and B220+ gated cells (middle and right FACs panels) are shown. (TIF) [file pgen.1008960.s004.tif]

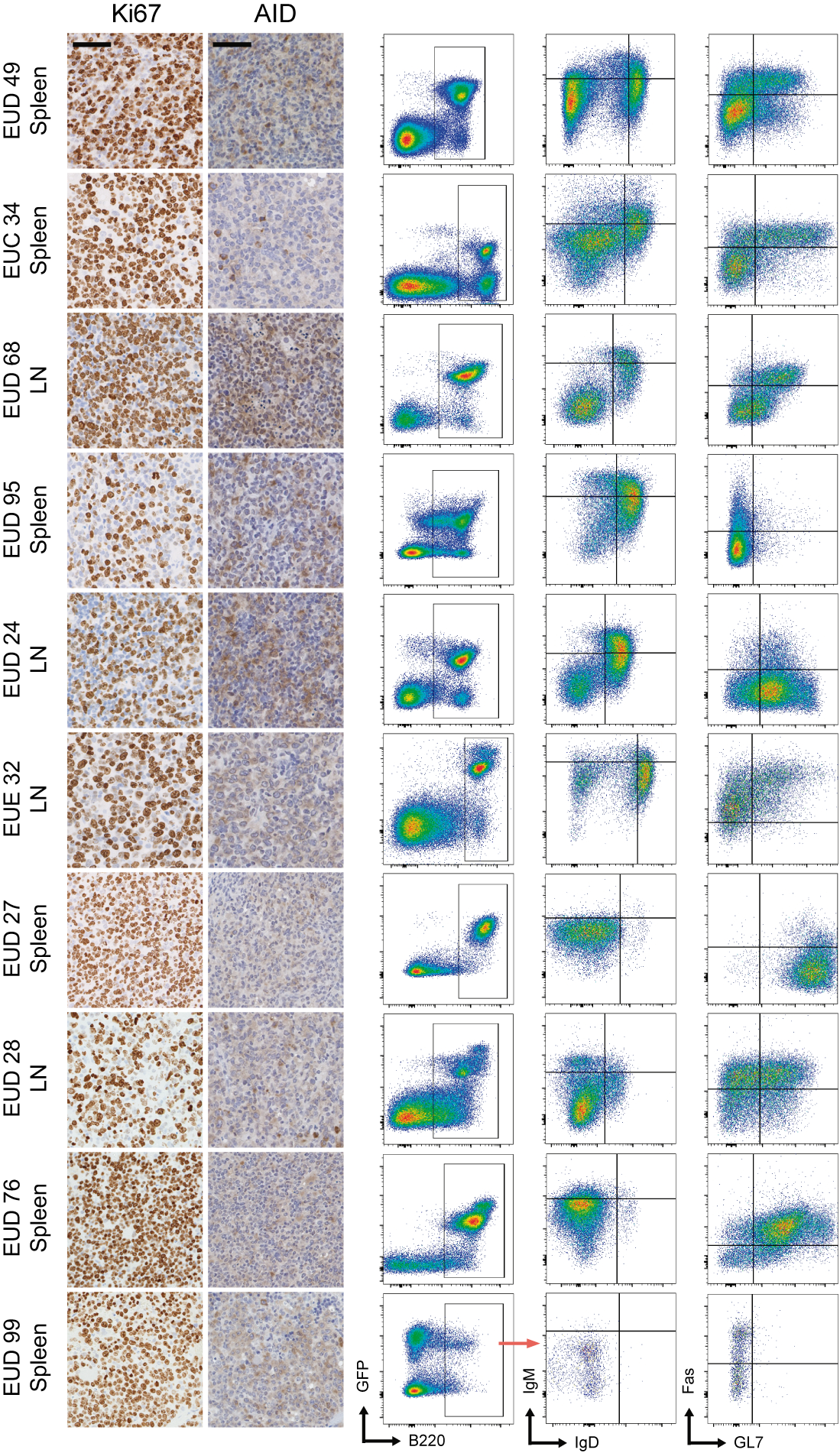

Supplement: S5 Fig — Magnification for Ki67 and AID IHQ images is 40x. Scale bar is 50μm. Total live cells (left FACs panel) and B220+ gated cells (middle and right FACs panels) are shown. (TIF) [file pgen.1008960.s005.tif]

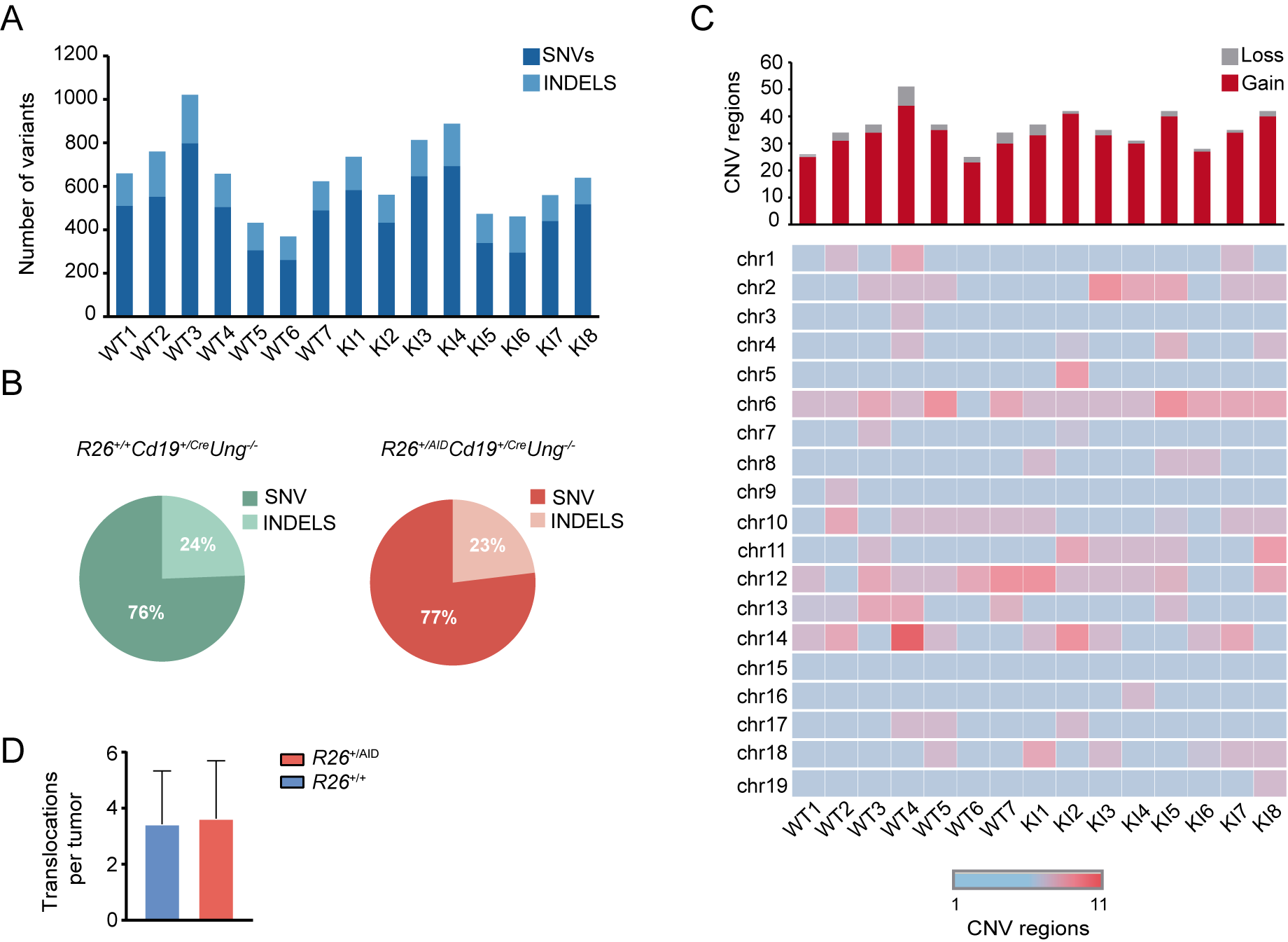

Supplement: S6 Fig — (A) Total number of SNVs and INDELS identified in each of the 15 tumors analyzed. (B) Average proportion of SNVs and INDELs found in R26+/+Cd19+/CreUng-/- and R26+/AIDCd19+/CreUng-/- tumors. (C) CNV analysis of tumors from WES data. Heatmap of the genomic distribution of CNV alterations in R26+/+Cd19+/CreUng-/- and R26+/AIDCd19+/CreUng-/- tumors. Upper panel depicts number of CNV regions per tumor, with colors encoding copy number gain (red) or loss (grey). (D) Number of translocations identified in R26+/+Cd19+/CreUng-/- and R26+/AIDCd19+/CreUng-/- tumors by Manta analysis of WES data (two-tailed t-test, p = 0.852; tumor vs healthy tissue, see methods for details). (TIF) [file pgen.1008960.s006.tif]
