# Supplementary material for: Interplay between UNG and AID governs intratumoral heterogeneity in mature B cell lymphoma
Source: PLoS Genet. 2020 Dec 23;16(12):e1008960. doi: 10.1371/journal.pgen.1008960 (PMC7790409; doi:10.1371/journal.pgen.1008960)
Supplement: Attachment — Submitted filename: Point-by-point_AR3.docx [file pgen.1008960.s013.docx]

**Delgado et al. MS# PGENETICS-D-20-00995**

**POINT-BY-POINT REPLY TO REVIEWERS’ CONCERNS**

**Reviewer #1**

Delgado et al. showed that a conditional knock-in of AID at the Rosa26 locus, when turned on in B lineage, was insufficient to generate B cell lymphoma. However, if combined with an UNG deficiency, B cell transformation is readily detected. Whole exome sequencing shows elevated somatic hypermutation in these tumors with increased subclonal variability and a wider range of oncogenic variants. These data reaffirm the mutagenic (oncogenic) potential of AID-generated uracils and provide direct evidence that UNG safeguards genomic integrity (at least partly) against AID’s DNA mutator activity. The experimental design is sound and the conclusions are supported by the data. This study should be of interest to a wide range of audience.

We are thankful for the reviewer´s positive assessment of our work. His/her comments have been addressed as follows,

1. Although elevated mutations were seen in R26+/AID Cd19+/cre Ung-/- tumors, whether or not these mutations are drivers for tumorigenesis is not known. The authors need to at least acknowledge that.

We agree with the reviewer that our study does not address the functional impact of the detected mutations as drivers for the tumorigenesis. Identifying true driver mutations is not straightforward and traditionally, recurrence of mutations across cancer patients has been used as an approximation to identify cancer driver mutations. Thus, we have carefully avoided the use of the “driver” terminology in our paper and mention instead “cancer-associated genes”. Despite this acknowledged limitation, we now include Table S5, which provides a list of genes mutated in our WES analysis that are considered drivers (according to IntOgen, see methods) or are recurrently mutated in B cell lymphoma. Within the latter, we can highlight EEF1A (mutated in 9% of FL, 10.1073/pnas.1501199112), Fas (mutated in 21% of DLBCL, 0.1182/blood-2008-12-194852) or Myd88 (mutated in 29% of ABC-DLBCL, 10.1038/nature09671), among others (Table S5). Therefore, while we cannot ascertain the actual functional impact of these mutations, we believe this analysis provides a good approximation to this issue.

2. Page 10, Ln 6. The mutation frequency analysis was restricted transitions at G/C pairs. The rationale for this restriction is not given. This seems unfair, especially without knowing whether tumorigenesis is driven only (or mostly) by these transition mutations (point #1).

We acknowledge that this point must be justified and apologize for our lack of clarity. In the absence of Ung, transversions are severely impaired (10.1016/S0960-9822(02)01215-0 and Figure 3 of our manuscript). Also, we have seen that off-target AID activity is mainly focused on G/C, rather than A/T nucleotides, whose average mutation rate is very close to our detection sensitivity (doi.org/10.1084/jem.20171738). Thus, analysis of only G/C transitions covers the vast majority of AID off-target mutations in this model, while reducing the noise signal. This point has now been explained on p10. Regarding whether tumorigenesis is driven only (or mostly) by these G/C mutations, we do not have a definitive answer, as explained above. However, most of the high impact mutations that we have detected are transitions at G/C (we now include Table S3 with the description of all variants detected in our WES analysis), probably reflecting that G/C transitions are indeed the overwhelming majority of AID-induced mutations in this model.

Minor points:

1. There is a recent study showing that GC might not be the dominant site for CSR (PMID: 31375460), the authors might want to consider modifying the introduction (Page 3), unless they hold a different view of that study.

We thank the reviewer for bringing up this point that we had overlooked. The intro has been slightly modified so that CSR is not defined as a GC event.

2. It has been reported that UNG protects against AID-induced telomere loss (PMID: 27697833). Although this may lead to B cell senescence instead of transformation, one cannot rule out the possibility that somehow telomere stress contributes to genome instability and oncogenesis. A brief discussion will be beneficial.

This is certainly an important point to discuss. We now refer to this article as well as to a very recent work from the same lab (10.1093/narcan/zcaa019), both in the introduction (p4) and in the discussion (p13) sections.

**Reviewer #2**

Thank you for the opportunity of reviewing the manuscript entitled: “Interplay between UNG and AID governs intratumoral heterogeneity in mature B

cell lymphoma” by Delgado et al.

The manuscript aims at addressing the contribution of AID in the process of lymphomagenesis with particular focus to the induction of SHM in lymphoma cells.

The Authors generated a new model of conditional supra-expression of AID selectively in B cells by knocking-in AID in the Rosa26 locus.

They observe lack of increase lymphoma formation with supra-expression of AID (probably achieving a lower over-expression than in other models of AID transgenesis) and absence of co-operation by p53 mutations. In contrast, a combined deficiency of UNG associated with AID supra-expression induces lymphoma formation in a subset of mice.

By characterizing the mutational patterns of these lymphoma, the Authors find that supra-expression of AID together with lack of UNG induces higher SHM rates in lymphomas, associated with higher subclonal heterogeneity of mutations.

Overall, the work is well presented and clearly written. Data are well organized, the design of the experiments is sound, the conclusions are supported by the data provided.

However, novelty is limited by the fact that most of the data are predictable from the current knowledge in the field, including the increased SHM rate when AID is coupled with UNG deficiency and the fact that UNG deficiency induces B cell lymphoma

We appreciate very much the reviewer’s positive assessment of our manuscript. We acknowledge that we had not fully convey the novelty of our work in our original manuscript. Now we have done so in responding to the reviewer’s comments (see below) as well as with additional context in the introduction section (p4) and further discussion (p13)

Major comments:

- the assumption that chromosomal translocations do not play a role in R26+/+-Cd19+/cre-Ung-/- and R26+/AID-Cd19+/cre-Ung-/- lymphomas has to be experimentally verified in this work. While it is established that UNG deficiency reduces chromosomal translocations driven by AID (as published in previous work by the corresponding Author), yet it is unclear whether translocations are completely abrogated or just reduced in UNG deficiency B cells, in particular when AID is overexpressed. The Authors should evaluated their tumors for the presence at least of c-myc translocations/amplifications.

We agree with the reviewer that this is an interesting point and that the absence of chromosome translocations in B cell lymphomas from Ung deficient mice has not been directly tested in our work. Unfortunately, the experimental groups used in our WES analysis do not include Ung proficient mice, as we are only assessing the impact of AID overexpression on mutagenesis by comparing R26+/+ Cd19+/cre Ung-/- versus R26+/AID Cd19+/cre Ung-/- lymphomas. However, we can tell whether AID supra expression increases chromosomal translocations in an Ung-/- background, the rationale being that if there was a detectable frequency of AID-dependent translocations in Ung-/- lymphomas, it would be expected to increase in the presence of excess AID. Conversely, if AID supra expression did not increase the translocation frequency in Ung-/-, the results would indicate that translocations are not responsible for the lymphoma differences observed between the R26+/+ Cd19+/cre Ung-/- and R26+/AID Cd19+/cre Ung-/- groups.

Our previous analysis of indels and copy number variation (CNV) shown in Figure S6 already suggested this latter hypothesis.

To directly address the reviewer’s point, we have now performed a complete analysis of chromosome translocations on our WES data using the Manta software (new Figure S6D and Table S4). We have found that indeed AID overexpression does not increase the translocation frequency of Ung-/- lymphomas. It can seem puzzling, though, that we typically find 2-4 translocations per mouse in the absence of Ung. These translocations could be AID-independent and thus generated in a pathway that does not require Ung either. Alternatively, this low translocation frequency could represent background detection of reads with fused transcripts. Unfortunately, we cannot distinguish between these two possibilities. Despite this limitation, our data indicate that the lymphoma alterations triggered by AID supra-expression are not due to an increase in translocation frequency, but rather, to an increase in SHM frequency. This in turn is consistent with previous studies showing that AID activity does not promote double strand breaks in the absence of UNG (10.1038/nature04495, 10.1038/nri.2016.2)

- It is unclear which are the criteria the Authors use to distinguish lymphoma FL-like or DLBCL-like phenotype. This distinction needs to be clearly explained and supported by data, otherwise it could be misleading because typically DLBCL carry more mutations than FL. Since R26+/AID-Cd19+/cre-Ung-/- lymphoma show increased and broader SHM, the expectation is of an increased and not reduced incidence of DLBCL in these mice. Can the Authors explain?

We apologize for this superficial lymphoma definition. Lymphomas were classified by our pathologist based on markers and morphological criteria, and he found that R26+/AID;Cd19+/cre;Ung-/- lymphomas were generally conformed of smaller cells than did lymphomas from R26+/+;Cd19+/cre;Ung-/-. This characterization has been expanded in our revised manuscript (new Figure 4E, new Figure S4 and S5). Classification of mouse B cell lymphomas is not a faithful parallel of human lymphomas, and molecular definitions, including information on mutational load, is relatively scarce. Thus, according to the Bethesda proposals (10.1182/blood.v100.1.246, 10.1177/0192623315587922) for classification of lymphoid neoplasms in mice, the lymphomas found in R26+/AID Cd19+/cre Ung-/- mice would be morphologically compatible with the definition of Follicular B cell Lymphoma (FBL), as well as small B cell lymphoma. However, we need to stress again that such classification does not include an assessment of tumor mutations, and therefore, whether the mouse FBL and DBCL recapitulate their human counterparts in this regard is currently unknown. In any case, we fully agree with the reviewer that using this nomenclature can be misleading, and have therefore renamed DLBCL-like and FL-like as large B cell (LB) lymphoma and small B cell (SB) lymphoma.In addition, we now specify in the Methods section the criteria used by our pathologist to make this classification.

**Minor comments**

- histology images should contain scale bars

Scale bars have been included

- reference 10 and 54 are the same

Duplication has been eliminated

**Reviewer #3**

Delgado et al generated mice that conditionally overexpress AID to test its contribution to B cell lymphoma development and evolution. AID o/e in the hematopoietic lineage, or from early B cell development is insufficient to affect mouse survival, even when combined with Tp53-/-. However, combined with Ung-/-, AID o/e from early B cell development results in increased lymphoma incidence compared to control Ung-/- mice. The lymphoma histopathology and GC marker expression profile in the Ung-/- R26AID are different from the Ung-/- but still mature B cell lymphoma. More importantly, WES showed that AID o/e in the absence of Ung increased heterogeneity in the tumoral clone with a clear AID mutational footprint. Mutations at genes that are often found mutated in human B cell lymphoma are also clonally mutated here, but they also analyze subclonal mutations, evidencing ongoing AID activity as a source of intratumor heterogeneity. They then focus on cancer-related genes, which are mutated in both mouse lines, but the AID o/e bears mutations in some additional genes.

This manuscript experimentally demonstrates that AID creates intratumor heterogeneity. While this might have been suspected, previous experimental systems could only show broad correlations of mutation signatures and did not analyze clonal and subclonal dynastic relationships. The overexpression system is in a way artificial but likely the only viable option to test this (see below). In addition, it allows them to better make the point that AID by itself is insufficient for lymphomagenesis. Since AID expression is well associated to poor outcome in human B cell neoplasms, the ITH demonstrated here acquires further relevance.

This is very good work, clearly written and technically sound. The data is solid, correctly analyzed, and very well discussed. I support publication. I have several question and comments that could be useful to address in the final manuscript, mostly to satisfy my curiosity.

We are delighted to read the reviewer’s positive evaluation of our work. We address below the reviewer’s specific questions.

The observation that AID overexpression does not lead to more CNV than endogenous AID seems worth emphasizing. This is a nice indication that most deletional / recombinogenic activity of AID is via UNG. If I am not mistaken, this has so far been inferred from the analysis of specific translocations, but not at the genomewide level. This could be emphasized in the discussion since it is important for their conclusion that point mutations by AID can accelerate lymphomagenesis.

We thank the reviewer for pointing this out. Indeed, the role of UNG in the recombinogenic outcomes of AID activity had never been assessed genome wide and that this is a novel and important point of our study. To stress this concept, we have directly analyzed the presence of chromosome translocations from our WES data using Manta software. In agreement with our copy number variation (CNV) and indel analysis (Figure S6A-C), we have also found that AID excess does not increase the rate of chromosome translocations in UNG deficient tumors (new Figure S6D). Although we could not assess translocation frequency in UNG proficient mice (since those groups of mice were not included in our ageing study), our data strongly indicate that the lymphoma alterations triggered by AID excess are due to an increase in SHM frequency. Following the reviewer’s suggestion, we have now emphasized this in our discussion.

This work compares tumors in Ung deficient mice that express two different doses of AID. The perfect but impossible control would have been similar tumors without AID, which just do not happen. This point could be made in the discussion for the sake of people not in the field.

This is a very important point. Unfortunately, an Aicda-/-Ung-/- control group was not included in our study. However, a very recent work by the di Noia lab has directly assessed lymphoma generation in Ung deficient mice in the absence of AID (Safavi et al 10.1093/narcan/zcaa019). Unexpectedly, they find that AID deficiency by itself makes mice prone to develop lymphoid hyperplasia, which leads to a decreased survival of Aicda-/- mice compared both to wild type and to Ung-/- mice. Thus, as pointed out by the reviewer, this control would have not been possible in our study. The Safavi et al work is now discussed in our manuscript.

I agree with their interpretation that moderate AID o/e may have partially contributed to avoid the negative regulation seen with transgenes and could explain the lack of tumors in wt or p53-/- background. Thus, it would be best to actually provide an estimate of how much more AID protein and activity is there in mature B cells in the R26AID compared to endogenous levels. AID protein could be quantified from the WB. The increase in IgG1 in vitro and Smu mutation data suggest ~2-fold but it may not represent activity off target. I guess the number of mutations at WRC could also be used as a proxy for off target activity, which seems to come about to 2-fold as well?

AID levels indeed seem to have an important role in determining AID’s oncogenic impact under different genetic backgrounds; as yet another intriguing and complex point of AID activity. Following the reviewer’s suggestion, we have now quantified AID protein in normal B cells by western blot, and found that activated R26+/AID cells have roughly x1.6 AID protein than activated R26+/+ cells (new Figure 1C). This increase is comparable to our qRT-PCR data in activated cells (Figure 1A, 1.3 fold). Likewise, the rate of CSR to IgG1 (Figure 1D, 1.5 fold) and our mutational data in germinal center B cells (Figure 3A, 1.2 fold in Ung+/- background, 1.6 fold in Ung -/- background) all seem to suggest a similarly mild increase. Thus, despite the variable sensitivity and diversity of readouts, in general we detect that the excess of AID levels and activity in activated/GC R26+/AID B cells is modest and do not reach the two-fold difference, compared to control mice.

I am also curious about the level of AID in the final tumors. The IHC in Fig 4D would suggests some heterogeneity between R26AID tumors in either the level of AID observed in the lymphoma cells or proportion of cells expressing AID. Is there any way to quantify this?

This would definitely be an interesting point to address, especially in the context of the recent data by the di Noia lab showing that UNG protects the fitness of AID-expressing B cells (Safavi et al 10.1093/narcan/zcaa019) We have now performed a more extensive study of AID expression in tumors from R26+/AID Cd19+/cre Ung-/- and R26+/+ Cd19+/cre Ung-/- mice (new Figure S4 and S5). Unfortunately, quantification of protein levels by IHC is complex, and AID stainings are not particularly clean. We did attempt to quantify the level of AID staining with ImageJ, but we believe the data were not reliable enough. Thus, we have measured the proportion of cells expressing AID using Tissuemorf software (VisioPharm), which quantifies areas of positive staining in the whole tissue section (Figure R1). However, we need to note that in these quantifications, the negative area includes not only tumoral cells that are negative for the staining but non-tumoral cells as well. This makes the interpretation of this evaluation not straightforward. Keeping these limitations in mind, our results show that AID expression was heterogeneous in both groups of mice, with no significant differences between them (Figure R1). One could suggest that AID expression in R26+/+ mice is more widespread, with a group of mice displaying a higher proportion of AID+ cells. However, this result would need confirmation before venturing an interpretation.

**Figure R1.** Quantification of AID staining in tumor samples. Tumoral spleens or lymph nodes from R26^+/+^ Cd19^+/cre^ Ung^-/-^ and R26^+/AID^ Cd19^+/cre^ Ung^-/-^ mice were formalin-fixed and embedded in paraffin and stained with anti-AID antibodies (Figure 4, S4 and S5). Images were digitized and then quantified using Tissuemorf (VisioPharm). Graph shows the proportion of AID stained area after subtracting the background signal, as obtained with anti-rat secondary antibody staining in each tumor.

In fig. 3, the authors show that mutation at the Smu is higher in UNG-/- cells when AID is o/e. However, endogenous AID barely increases mutations in UNG ko GC cells. This would suggest that UNG is not much of a break against endogenous AID mutagenic activity. Yet, Ung-/- tumors are said to show AID signature. It is not clear how this was determined. Does the total frequency of mutation differ between the two groups? Do mutations at C/G or at WRC happen at a higher than expected frequency in the R26+/+ Ung-/- tumors? It would be best to clarify this.

The reviewer is right. Figure 3A (right) shows that in an Ung-/- background, AID over expression increases mutations, and conversely, that in a context of AID over expression, Ung deficiency increases mutations. In contrast, Ung deficiency does not promote a significant increase in mutation frequency at endogenous AID levels. In the latter case we only detect a trend, possibly because of the contribution of C/G transversions and mutations in A/T in Ung proficient cells. Indeed, the frequency of C/G transitions is increased by Ung deficiency in B cells with endogenous levels of AID (Figure 3B, right). This point has been clarified in the text (p8).

For the tumoral context, we cannot directly compare the AID signature between Ung proficient and deficient tumors because we lack Ung proficient mouse groups in our WES study. Thus, we cannot tell whether mutations at C/G or WRC occur at a higher than expected frequency in the R26+/+ Ung-/- tumors as compared to Ung proficient tumors. Instead, we can tell that in R26+/+ Ung-/- tumors, transition frequency at WRC/GYW is higher that total transition frequency at C/G (Figure 5A, striped blue bar versus solid blue bar), indicating AID hostspot focusing. Likewise, we observed that, in Ung-/- tumors, AID overexpression increased the frequency of transitions at C/G (Figure 5A, solid red bar versus solid blue bar) and enhanced the AID signature (Figure 5A, striped red bar versus solid red bar). We have clarified this point (p10) and p values have been added to the figure legend.

The previous point has implications for the etiology of B cell lymphoma found in R26AID but also in the control Ung-/- mice. Does AID contribute to lymphomagenesis in both? Both are mature but different, at least histologically. It would be useful to have the proportion of lymphoma in each line that were positive for Pax5, B220, GL7, etcl; to better assess how robust is the change in the type of lymphoma. Is there any indication that tumors from R26AID are more aggressive (i.e. more Ki67+?). Can they speculate on the cause? AID would be expressed very differently, over the whole development in R26AID versus only after activation in the controls. Maybe each lymphoma type derives from a different mature B cell type? Or is it just a reflection of mutations by the o/e AID targeting genes that are less frequently targeted by endogenous AID?

The reviewer raises interesting questions regarding the lymphomagenic mechanisms and cell origin for R26+/+ Cd19+/cre Ung-/- and R26+/AID Cd19+/cre Ung-/- tumors. Unfortunately, at the moment we can only speculate on these issues beyond our observations that the tumors are morphologically different and that survival is decreased in R26+/AID Cd19+/cre Ung-/- mice.

We have now extended tumor characterization and have included in our manuscript additional flow cytometry data (Figures S4 and S5, Figure 4E). These analyses may suggest a higher proportion of tumors expressing the Fas or GL7 GC markers, which could have been a hint of a distinct cell of origin -closer to a GC cell-; however, 1) this trend is not significant, and 2) these markers may not bear etiological relevance. Likewise, we have stained our tumor collection with anti-AID and anti-Ki67 antibodies (Figures S4 and S5) and have quantified these stainings using Tissuemorf. As discussed above, the results of this quantification are confounded by the proportion of tumoral cells in the section that is analyzed; thus, the negative fraction of tissue comprises both non-tumoral cells and tumoral cells with low or negative expression of the marker. Since we cannot ascertain the fraction of non-tumoral cells, these analyses are hard to interpret. Indeed, quantification of Ki67 staining area (Figure R2) shows a similarly broad range of positive cells both in R26+/+ Cd19+/cre Ung-/- and R26+/AID Cd19+/cre Ung-/- tumors, which very likely reflects the proportion of tumoral cells rather than the aggressiveness of the tumor.

**Figure R2.** Quantification of Ki67 staining in tumor samples. Tumoral spleens or lymph nodes from R26^+/+^ Cd19^+/cre^ Ung^-/-^ and R26^+/AID^ Cd19^+/cre^ Ung^-/-^ mice were formalin-fixed and embedded in paraffin and stained with anti-Ki67 antibody (Figure 4, S4 and S5). Images were digitized and then quantified using Tissuemorf (VisioPharm). Graph shows the proportion of Ki67 stained area.

Tumor characterizations are now expanded in new Figures S4, S5 and 4E has been clarified in the text (p9)

The reviewer raises the interesting hypothesis that R26+/AID Cd19+/cre Ung-/- tumors could arise from an earlier cell of origin -given the earlier onset of R26AID expression, compared to the endogenous AID. This is however hard to ascertain, unless one could use a genetic tracer strategy to identify cells having gone through a particular differentiation stage.

Finally, regarding whether AID can contribute to lymphomagenesis in R26+/+ Cd19+/cre Ung-/- mice, we believe that the AID hotspot focusing we found in R26+/+ Cd19+/cre Ung-/- tumors (Figure 5A) suggests that AID-dependent off-target mutations are at place in the context of endogenous AID expression, although they occur at a higher frequency in R26+/AID Cd19+/cre Ung-/- mice. In addition, R26+/+ Cd19+/cre Ung-/- and R26+/AID Cd19+/cre Ung-/- tumors share a big proportion of mutated cancer-related genes. Based on these observations we favor the hypothesis that the difference in lymphoma development in these two groups of mice are the consequence of an increase mutation load in R26+/AID Cd19+/cre Ung-/- tumors of genes that are less frequently targeted by endogenous AID.

These latter points are now briefly discussed in our revised manuscript (p12-13).

A further point raised by the previous comments is whether UNG is a tumor suppressor in humans. It is clearly so in the mouse and UNG deficiency is useful here to prove the point about ITH by SHM, but to my knowledge, UNG has not been singled out as a commonly mutated gene in human B cell lymphoma. The authors mention that UNG loss has been found in human lymphoma. It would be best to qualify this. How frequent is UNG loss of function?

This is an important point. L Staudt reported UNG mutations (Schmitz et al, 10.1056/NEJMoa1801445, Supplementary Appendix 2, Figure R3), although at a low frequency (0.4% of DLBCL). This low incidence probably reflects that UNG has a weak tumor suppressor activity. This is indeed consistent with observations in mice by us (here) and others, in that UNG loss, even if combined with AID supraexpression, has only a mild impact on survival. This low incidence is specifically mentioned in the text (p13).

**Figure R3.** Incidence of UNG loss of function mutations in B cell lymphomas.

Extracted from 10.1056/NEJMoa1801445

Have all data underlying the figures and results presented in the manuscript been provided?

Large-scale datasets should be made available via a public repository as described in the PLOS Genetics data availability policy, and numerical data that underlies graphs or summary statistics should be provided in spreadsheet form as supporting information.

Reviewer #1: Yes

Reviewer #2: Yes

Reviewer #3: No: most numerical data is about the analysis of mutations from WES; so numbers may be covered by the data deposited. but there is no excel file with the numbers for plots shown in other figures (I apologize if I missed it)

We apologize to the reviewer for failing to include supplementary Tables S1-4 (as originally cited in the text). We have now amended this mistake and have included an additional supplementary table with our translocation analysis. The information contained in this material is as follows:

Table S1. Whole Exome Sequencing (WES) metrics of the 16 samples analyzed.

Table S2. Tumors samples analyzed by WES

Table S3. Table S3. Variants identified in tumors.

Table S4. Translocation analysis of tumors

Table S5. List of cancer-related genes mutated in WT and/or KI tumors

In addition, raw sequencing data is deposited in GEO.
